# Successful Treatment of Nivolumab Plus Ipilimumab After Progression on Durvalumab Plus Tremelimumab in Advanced Hepatocellular Carcinoma: A Case Report

**DOI:** 10.7759/cureus.97530

**Published:** 2025-11-23

**Authors:** Akinori Sasaki, Rika kimura, Risa Okamoto

**Affiliations:** 1 Gastroenterology, Tokyo Bay Urayasu Ichikawa Medical Center, Urayasu, JPN

**Keywords:** advanced hepatocellular carcinoma, durvalumab plus tremelimumab, immune checkpoint inhibitors, immune‑mediated adverse events, nivolumab plus ipilimumab

## Abstract

Dual immune checkpoint inhibitor (ICI) regimens used to treat advanced hepatocellular carcinoma (HCC) include durvalumab plus tremelimumab and nivolumab plus ipilimumab, but optimal sequencing after progression on the former remains unclear, especially when VEGF-targeted options are limited by comorbidities. A 70-year-old man with chronic kidney disease and proteinuria had unresectable recurrent HCC. He underwent proton beam therapy followed by salvage surgery; however, he subsequently developed recurrent disease that was not amenable to further surgical resection. Durvalumab plus tremelimumab was chosen as first-line systemic therapy owing to contraindications to bevacizumab and tyrosine kinase inhibitors. After two months, CT showed progressive nodal disease with new right-sided pleural dissemination and malignant effusion; the response was progressive disease per Response Evaluation Criteria in Solid Tumors (RECIST) v1.1. Therefore, nivolumab plus ipilimumab was commenced as subsequent systemic therapy. After two cycles, he developed grade 2 dermatitis, which was managed with topical corticosteroids. A CT scan revealed a reduction in nodal and pleural lesions, as well as a significant decrease in effusion. At three months, grade 3 immune-mediated colitis required hospitalization; intravenous corticosteroids followed by infliximab led to recovery. ICIs were not reintroduced, and no radiologic progression was observed during the subsequent two months.

## Introduction

Hepatocellular carcinoma (HCC) accounts for the majority of primary liver cancers and remains a leading cause of cancer death worldwide; in many regions, including Japan, the etiologic profile is shifting from viral hepatitis to alcohol-related and metabolic liver disease [1]. Many patients are diagnosed at an advanced stage or experience recurrence after locoregional therapy [1]. Immune checkpoint inhibitors (ICIs) have reshaped the systemic treatment landscape in cancer treatment. In the first-line setting, atezolizumab plus bevacizumab improved survival versus sorafenib and became a standard option for unresectable disease [2]. Subsequently, dual ICI therapy with a single priming dose of tremelimumab combined with durvalumab (the STRIDE regimen) also demonstrated an overall survival benefit and has since been adopted in routine practice, including in Japan [3]. More recently, nivolumab plus ipilimumab showed superior overall survival versus tyrosine kinase inhibitor (TKI) comparators in untreated unresectable HCC, further expanding ICI-based strategies in the frontline setting [4]. Collectively, these findings indicate that three regimens are available as first-line options for advanced HCC; however, because no head-to-head randomized trials have directly compared them, it remains uncertain which treatment is superior. Furthermore, after failure of first-line therapy, it also remains unclear whether patients are better served by sequencing to another ICI-containing regimen or by switching to a TKI (e.g., lenvatinib); robust comparative data are lacking.

Durvalumab plus tremelimumab and nivolumab plus ipilimumab are dual ICI therapies; tremelimumab and ipilimumab are both anti-CTLA-4 antibodies. In contrast, durvalumab targets PD-L1, whereas nivolumab targets PD-1. Generally, PD-1 inhibitors prevent PD-1 from engaging with both PD-L1 and PD-L2, whereas PD-L1 antibodies do not interrupt PD-1-PD-L2 signaling. This biologic distinction provides a rationale for sequencing to PD-1/CTLA-4 after PD-L1/CTLA-4 failure. Consistent with this biology, responses to PD-1 inhibitors after lack of benefit from PD-L1 antibodies have been reported in other tumor types [5, 6]. Clinically, such sequencing may be particularly relevant when VEGF-targeted therapy is contraindicated (e.g., clinically significant proteinuria or variceal bleeding risk), where a checkpoint-only strategy must be considered.

Here, we report a patient with advanced HCC and chronic kidney disease who experienced radiologic progression on durvalumab plus tremelimumab but subsequently achieved a meaningful response to nivolumab plus ipilimumab. To our knowledge, no prior published case in HCC has documented a radiologic response to PD-1/CTLA-4 blockade after progression on PD-L1/CTLA-4 blockade. We describe the clinical course, safety considerations in the setting of renal comorbidity, and the mechanistic rationale for sequential checkpoint inhibition after dual-ICI failure. This contributes case-level evidence to inform post-durvalumab plus tremelimumab treatment sequencing.

## Case presentation

**Figure 1:**
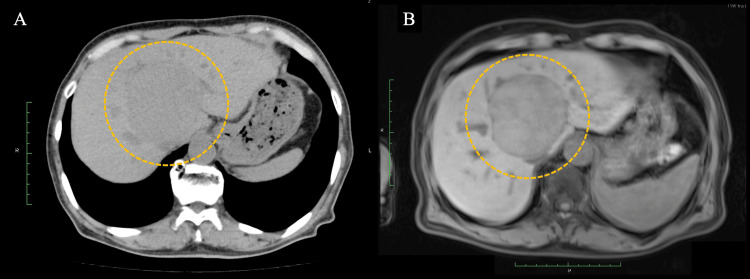
Images at diagnosis (A) Abdominal CT demonstrates a 70-mm hepatocellular carcinoma in hepatic segment 1 (S1) (dotted circle). (B) Abdominal MRI likewise showes a lesion at the same site on T1-weighted sequences. The disease was confined to a single lesion in hepatic S1 (dotted circle).

**Figure 2:**
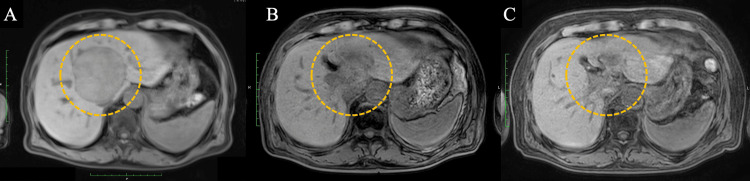
Serial imaging changes of hepatocellular carcinoma after proton beam therapy (A) Pre-treatment MRI shows a 70-mm hepatocellular carcinoma in hepatic segment 1 (dotted circle). (B) MRI at one year after proton beam therapy demonstrates lesion shrinkage with indistinct lesion margins (dotted circle). (C) MRI at two years after proton beam therapy shows complete disappearance of the lesion (dotted circle).

**Figure 3:**
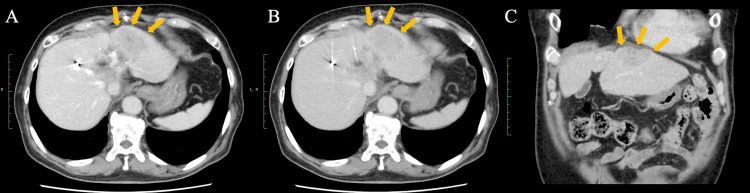
Imaging of recurrent hepatocellular carcinoma after proton beam therapy (A,B,C) Recurrent hepatocellular carcinoma is seen in the left hepatic lobe adjacent to the prior proton beam treatment site (arrow).

**Figure 4:**
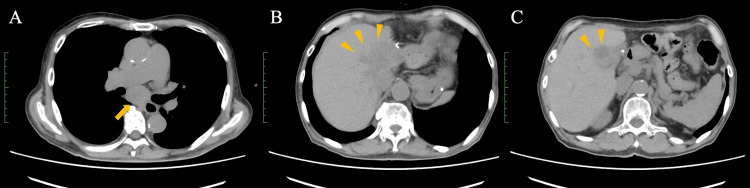
Recurrent hepatocellular carcinoma after surgery (A) Postoperative imaging demonstrates recurrent disease involving the mediastinal lymph nodes (arrow). (B, C) Additional images show intrahepatic recurrent metastases, indicating multifocal recurrence following surgery (arrowheads).

A 70-year-old Japanese man with a history of chronic kidney disease and proteinuria was diagnosed with HCC. The patient’s underlying liver disease was alcohol-related cirrhosis, and testing for the hepatitis B virus (HBV) and hepatitis C virus (HCV) was negative. Baseline imaging demonstrated a 70-mm lesion in hepatic segment 1. Due to its size and location, the tumor was deemed unresectable (Figure 1). Because of underlying chronic kidney disease and proteinuria, the patient was also deemed ineligible for transarterial chemoembolization and for cytotoxic chemotherapy. Accordingly, the patient underwent proton beam therapy and achieved a complete remission after treatment (Figure 2). He was subsequently monitored with routine surveillance using contrast-enhanced CT and MRI. However, two years after completion of proton beam therapy, local recurrence developed at the treated site, for which salvage surgery was performed (Figure 3). Although the lesion was completely resected, a follow-up CT scan at six months postoperatively demonstrated recurrent lymph node and intrahepatic metastases (Figure 4).

In parallel with the above course, the patient was diagnosed with unresectable, metastatic recurrent HCC. His performance status was initially good; however, because of chronic kidney disease with clinically significant proteinuria, anti‑VEGF treatment (e.g., bevacizumab) and TKIs such as lenvatinib were considered unsuitable. As durvalumab plus tremelimumab was approved and reimbursed in our jurisdiction, combination ICI therapy was selected as first‑line systemic treatment following a shared decision‑making discussion with the patient. He received the priming dose of tremelimumab with durvalumab, followed by durvalumab maintenance as indicated in the label.

**Figure 5:**
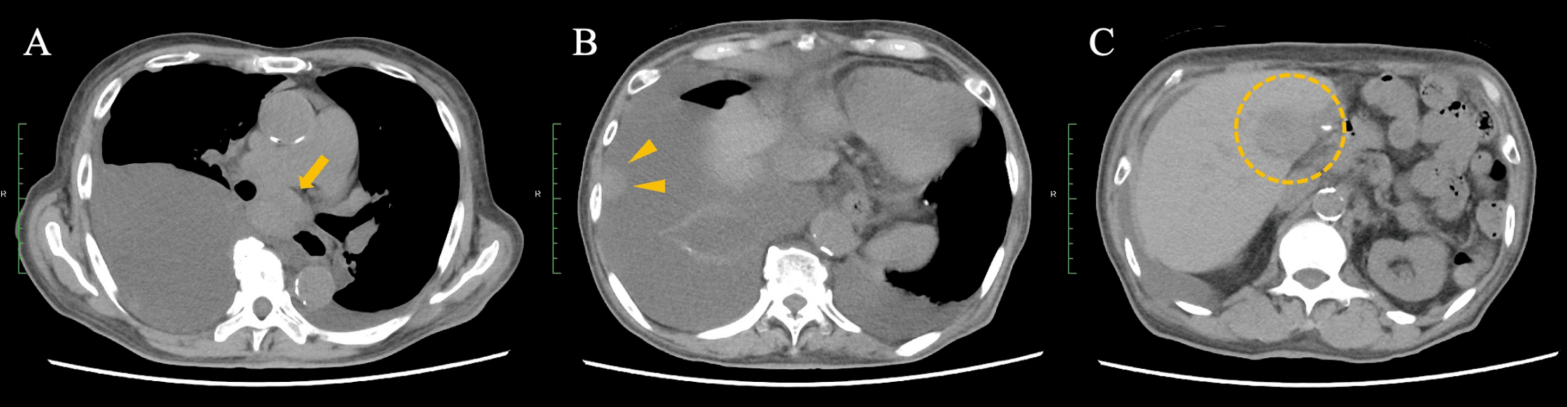
CT images obtained after two cycles of treatment with durvalumab plus tremelimumab (A) Chest CT shows enlargement of mediastinal lymph nodes with a new, large right pleural effusion (arrow). (B) Pleural dissemination is observed within the right pleural effusion (arrowheads). (C) Abdominal CT demonstrates interval enlargement of intrahepatic metastatic lesions (dotted circle). These findings warranted classification as progressive disease on durvalumab plus tremelimumab.

**Figure 6:**
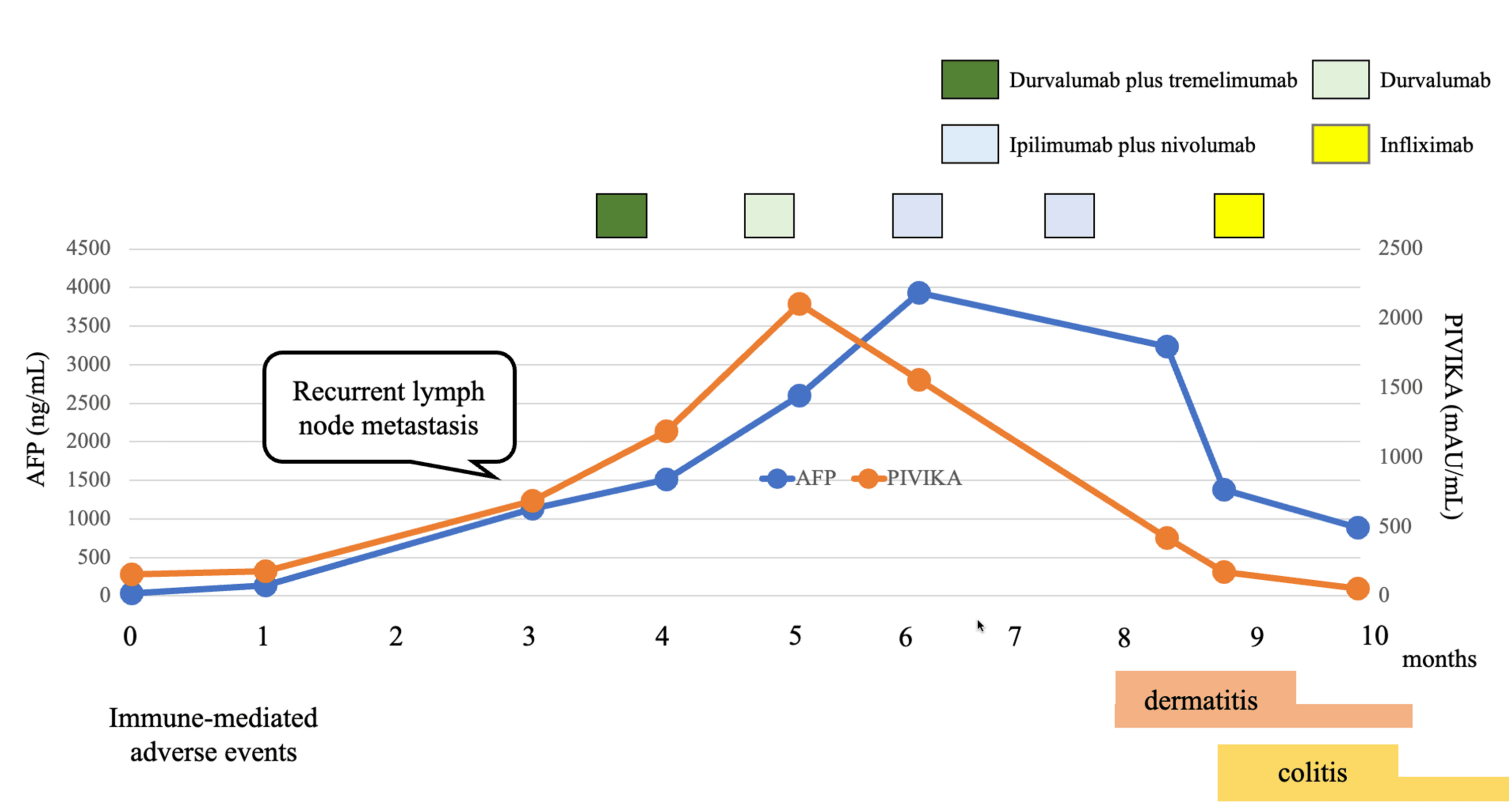
Course of treatment The figure summarizes the administration schedule of chemotherapy and biological agents, trends in tumor markers, immune-mediated adverse events, and the trajectory of Eastern Cooperative Oncology Group Performance Status. Although tumor markers continued to rise during durvalumab plus tremelimumab, they declined promptly after the switch to nivolumab plus ipilimumab. AFP: alpha-fetoprotein

A disease assessment CT scan performed approximately two months after treatment initiation demonstrated enlargement of nodal metastases together with new right‑sided pleural dissemination and malignant pleural effusion (Figure 5). Serum tumor markers were also elevated (Figure 6). The overall response was therefore classified as progressive disease, according to the Response Evaluation Criteria in Solid Tumors (RECIST) version 1.1 [7], and the patient’s performance status declined to 2. Due to contraindications to VEGF-targeted therapy and selected TKIs due to clinically significant proteinuria, the need for rapid disease control given symptomatic progression, and the patient’s preference following a shared decision-making discussion, nivolumab (1 mg/kg) plus ipilimumab (3 mg/kg) was initiated every three weeks as subsequent systemic therapy under regulatory approval in our country.

**Figure 7:**
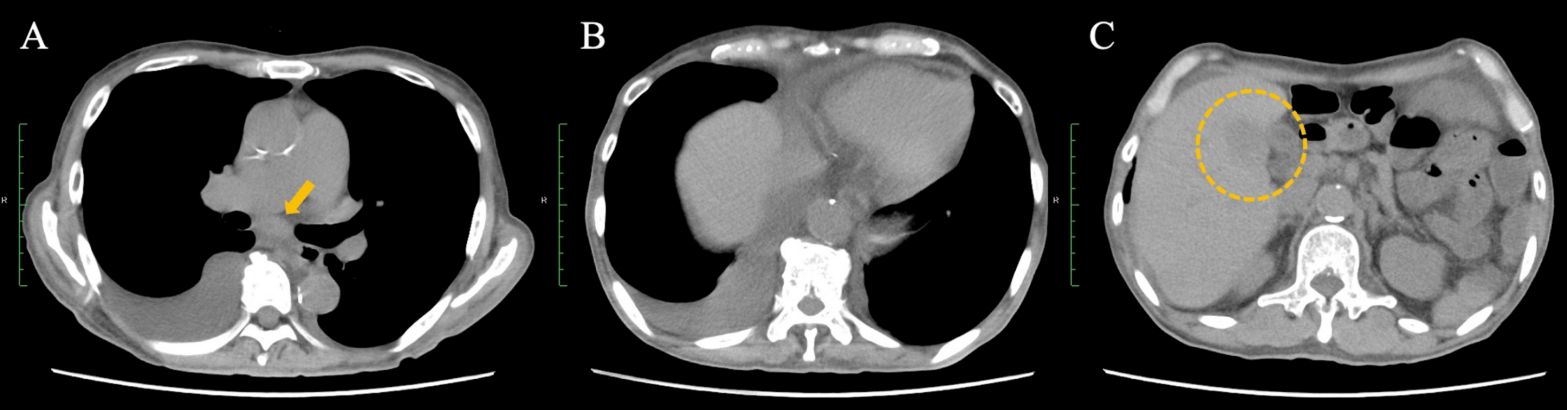
CT images obtained after two cycles of treatment with nivolumab plus ipilimumab (A, B) Chest CT shows a marked decrease in the right pleural effusion with a concurrent reduction in mediastinal lymph node size; pleural dissemination is no longer visualized (arrow). (C) Abdominal CT likewise demonstrates shrinkage of the intrahepatic metastatic lesions (dotted circle). The response to nivolumab plus ipilimumab was assessed as a partial response according to Response Evaluation Criteria in Solid Tumors (RECIST) version 1.1 [7].

After two induction cycles, the patient developed grade 2 immune‑mediated dermatitis, which improved with topical corticosteroids and symptomatic therapy, allowing treatment continuation. A CT scan performed about two months after starting nivolumab plus ipilimumab showed shrinkage of the involved lymph nodes and pleural implants, with a marked reduction in the right pleural effusion (Figure 7). Based on these imaging findings, the treatment response was determined to be a partial response according to RECIST v1.1 [7].

Approximately three months after initiation, he developed grade 3 immune‑mediated colitis and diarrhea requiring hospitalization. Intravenous corticosteroids were started (prednisone 1 mg/kg; 40 mg/day equivalent), but the response was suboptimal; infliximab 5 mg/kg was administered with subsequent clinical improvement. Prednisone was tapered to 20 mg/day, and the patient was discharged on hospital day 28. Since discharge, ICIs have not been reintroduced; on surveillance over the subsequent two months, there has been no radiologic evidence of disease progression (Figure 6).

## Discussion

This case demonstrates a clinically meaningful response to nivolumab plus ipilimumab after radiologic progression on durvalumab plus tremelimumab in advanced HCC. The observation is notable because clinically significant proteinuria and chronic kidney disease precluded VEGF‑targeted therapy and some TKIs, thereby limiting systemic treatment options. To our knowledge, there is no prior HCC report documenting a radiologic response to PD-1/CTLA-4 blockade after PD-L1/CTLA-4 failure. Our experience suggests that dual checkpoint blockade with PD‑1 and CTLA‑4 may retain antitumor activity after prior PD‑L1/CTLA‑4 exposure in selected patients.

The mechanisms underlying the observed response to nivolumab plus ipilimumab after failure of durvalumab plus tremelimumab remain uncertain; however, several hypotheses can be proposed. First, whereas durvalumab is an anti-PD-L1 antibody, nivolumab targets PD-1. Anti-PD-L1 antibodies restore antitumor immunity primarily by blocking PD-1-PD-L1 ligation on T cells, while anti-PD-1 antibodies prevent interactions with both PD-L1 and PD-L2, potentially resulting in broader T-cell reinvigoration and antitumor activity. Consistent with this concept, a meta-analysis in non-small cell lung cancer reported superior overall survival with anti-PD-1 therapy compared with anti-PD-L1 therapy [8]. Furthermore, emerging correlative evidence links PD-L2 expression with reduced responsiveness to anti-PD-L1 antibodies [9], whereas preclinical work indicates that PD-1 blockade retains antitumor activity in PD-L2-expressing tumors [10]. Taken together, these data suggest that a therapeutic switch from anti-PD-L1 to anti-PD-1 could extinguish PD-1 PD-L2-mediated inhibition and thereby foster renewed T-cell activity within the hepatic immune microenvironment [11, 12]. Second, the induction phase of ipilimumab plus nivolumab provides repeated CTLA‑4 blockade, in contrast to the single priming dose of tremelimumab in STRIDE, and may enhance de novo priming, expand tumor‑reactive clones, and reduce intratumoral regulatory T cells [3, 4, 13]. These distinctions offer a biologic rationale for attempting PD‑1/CTLA‑4 intensification after durvalumab plus tremelimumab failure.

From a practical standpoint, our patient showed a reduction in nodal and pleural disease after two induction cycles despite prior progression on durvalumab plus tremelimumab. Immune‑mediated adverse events (imAEs) (grade 2 dermatitis, grade 3 colitis) were managed according to contemporary guidance, including topical corticosteroids for dermatitis and systemic steroids followed by infliximab for steroid‑refractory colitis, with clinical recovery [14]. The occurrence of these imAEs following initiation of nivolumab plus ipilimumab suggests reactivation of T-cell-mediated immunity [15]. Although monoclonal antibodies generally do not require dose adjustment in chronic kidney disease, vigilant monitoring for immune‑mediated nephritis is essential [16].

The optimal strategy after progression on durvalumab plus tremelimumab remains unsettled. In practice, treatment selection should consider VEGF-related contraindications (e.g., clinically significant proteinuria or variceal bleeding risk), performance status, hepatic reserve (Child-Pugh class), disease tempo/burden, prior toxicities (including imAEs), and patient preference [17]. For patients in whom VEGF inhibition and certain TKIs are undesirable, re-intensification with PD-1/CTLA-4 may be reasonable, particularly when rapid disease control is needed and PS/organ function is preserved, supported by the activity of nivolumab plus ipilimumab in frontline trials and earlier-line cohorts [4]. Conversely, TKIs (e.g., lenvatinib, sorafenib, and cabozantinib) remain appropriate when comorbidities, toxicity profiles, or logistical considerations favor targeted therapy. However, comparative data defining the superior sequence after durvalumab plus tremelimumab are limited and are largely extrapolated from populations not treated with durvalumab plus tremelimumab [18]. Prospective, biomarker-informed studies are required to clarify optimal sequencing. For instance, in PD-L2-overexpressing HCC, post-STRIDE treatment with nivolumab plus ipilimumab could be expected to yield therapeutic benefit following nonresponse to durvalumab plus tremelimumab.

This report has limitations. Single‑patient observations cannot establish causality or generalizability, and the lack of correlative biomarkers (e.g., PD‑L2 expression, T‑cell receptor repertoire, and tumor mutation burden) prevents mechanistic confirmation. Moreover, the study is constrained by a short follow-up interval of about three months after nivolumab plus ipilimumab initiation, which should be acknowledged as a limitation. Nonetheless, our case contributes clinical granularity to an understudied sequence and may inform hypothesis generation. Future studies should include prospective comparisons of PD‑1/CTLA‑4 intensification versus TKIs after durvalumab plus tremelimumab and translational studies to identify predictive biomarkers and toxicity‑response correlates.

## Conclusions

In a patient with advanced HCC who experienced progression on durvalumab plus tremelimumab, nivolumab plus ipilimumab achieved a clinically meaningful response despite renal comorbidity that limited VEGF‑targeted options. At the last assessment, disease control was maintained approximately three months after PD-1/CTLA-4 initiation. This observation supports the hypothesis that dual PD‑1/CTLA‑4 blockade can retain efficacy after prior PD‑L1/CTLA‑4 exposure. Clinicians may consider sequential PD-L1 to PD-1/CTLA-4 blockade in carefully selected patients who are unsuitable for VEGF inhibition, with shared decision-making and close toxicity monitoring. Although single‑patient data cannot determine generalizability, our case provides a real‑world context for post‑durvalumab plus tremelimumab sequencing, and prospective biomarker studies are warranted to confirm PD-1/PD-L2 cross-resistance mechanisms.
